# Vanadium Compound Treatment Modulates MC3t3-E1 Osteoblast Function

**DOI:** 10.3390/ijms26178682

**Published:** 2025-09-05

**Authors:** Isabella K. Somera, Bryan Sosa, Jessica A. Cottrell

**Affiliations:** Department of Biological Sciences, Seton Hall University, South Orange, NJ 07079, USA

**Keywords:** osteoblasts, vanadium compounds, insulin mimetic, insulin signaling

## Abstract

Osteoblastogenesis plays a critical role in bone repair. Insulin and insulin-mimetic compounds, such as vanadium (IV) oxide acetylacetonate (VAC), have been reported to enhance bone healing in various models. This study aimed to evaluate the effects of vanadium compounds, VAC and vanadium (IV) oxide sulfate (VOSO_4_), on osteoblast proliferation and function. MC3T3-E1 pre-osteoblast cells were treated with insulin, ascorbic acid, and varying concentrations of VAC or VOSO_4_, and samples were collected at multiple time points over 21 days. We assessed cell proliferation, functional markers, and gene and protein expression. Our findings demonstrate that both VAC and VOSO_4_ stimulate MC3T3-E1 proliferation, increase calcium and proteoglycan deposition, and enhance phosphorylation of Protein Kinase B (Akt) over time. Gene expression analysis revealed that VAC treatment upregulated RUNX2, BGLAP, and TWIST2 at Day 7 compared to controls, with sustained expression patterns observed at Day 10. These results align with existing literature, supporting that VAC and VOSO_4_ promote osteoblastogenesis and may serve as effective adjuvants to accelerate bone regeneration during fracture healing.

## 1. Introduction

According to the Global Burden of Disease (GBD) study in 2019, an estimated 178 million new fractures occurred worldwide, with 25.8 million contributing to years lived with disability (YLDs) [1]. A subsequent 2021 GBD update reflected a similar trend, reporting 172.8 million fractures and 25.2 million YLDs [2]. Notably, fractures associated with low bone mineral density accounted for 9.81 million disability-adjusted life years (DALYs), representing a 121% increase since 1990 [1,2]. Advancing research in fracture healing is essential to improve recovery outcomes, reduce long-term disability, and develop targeted therapies that restore skeletal integrity more effectively across diverse patient populations.

Bone fracture repair is a multifaceted process that is regulated by biological and mechanical aspects. Recovery times can vary from a few weeks to several years, depending on the severity of the condition and individual patient factors [3]. Current therapeutic approaches such as cyclic axial micromovements, various forms of electromagnetic stimulation, and targeted pharmacological interventions have shown effectiveness in accelerating the healing process of cartilage and bone cells [4,5,6,7,8,9].

Successful fracture repair depends on the orderly activation and maturation of various tissue lineages over a tightly regulated timeline [4,5,10,11]. Following physical trauma, a hematoma forms at the fracture site, composed of blood and bone marrow cells that initiate a localized inflammatory response. Once the primary hematoma is established, cartilaginous tissue develops to form a soft callus, providing structural stabilization to the healing bone. During chondrogenesis, chondrocytes within the soft callus proliferate and undergo hypertrophy, accumulating calcium-containing granules as a result of the hypoxic microenvironment surrounding the fracture [4,5,10,11]. Once these calcium granules are exported to the extracellular matrix, they interact with phosphate to trigger mineral deposition. These mineralized deposits give rise to the hard callus, which reinforces structural stability at the fracture site. However, the hard callus alone does not fully restore the bone’s biomechanical integrity. Functional recovery requires a remodeling phase, during which the bone architecture adapts to mechanical loading and gradually regains the capacity for normal weight bearing [4,5,11,12,13]. In normal bone homeostasis, tumor necrosis factor-α (TNF-α) and interleukin-1 (IL-1) induce a cascade of events to recruit osteoclasts to resorb hard callus bone, while osteoblasts facilitate lamellar bone deposition, progressively restoring structural integrity and stabilizing the fracture site [11]. Specific pharmacological agents, including insulin, can modulate early callus cell proliferation, while others exert effects on the differentiation pathways of osteoblasts and chondrocytes.

The MC3T3 cell line, derived from mouse osteoblast precursors, serves as a reliable and accessible in vitro model for investigating osteoblast transcriptional regulation, owing to its consistent differentiation capacity under straightforward monolayer culture conditions [14,15,16,17]. Treatment with ascorbic acid (AA) enhances the expression of transforming growth factor (TGF)-β, osteopontin (OPN), and estrogen receptor (ER)-α in osteoblasts, all of which play essential roles in bone formation [18]. Bone formation occurs when osteoblasts are more active than osteoclasts. In a study involving ovariectomized mice, ascorbic acid (AA) was shown to preserve osteoblast differentiation markers (Runx2, BMP-2, osteocalcin, Osterix), decrease bone loss, and promote bone formation [6,19,20]. Fracture studies demonstrate that localized insulin treatment significantly promotes callus cartilage formation in both normoglycemic and diabetic rat models. A study conducted by Cornish et al. found that insulin injections directly increased indices of bone formation in the calvaria of normoglycemic adult mice [21]. Gandhi et al. [6] employed an intramedullary insulin delivery system to assess the impact of localized insulin on bone repair using a diabetic rat femur model. Their study showed that targeted insulin release at the fracture site effectively corrected the impaired healing typically seen in diabetes, restoring both early chondrogenic activity and later stages of callus development and mechanical integrity. Notably, overall healing was significantly compromised when systemic insulin levels declined, underscoring the importance of maintaining adequate local insulin concentrations at the fracture site [6]. While local insulin administration is capable of inducing osteoblast proliferation through activation of Akt signaling, complications related to managing blood-glucose levels are an important concern.

Consequently, researchers have explored insulin-mimetic compounds like vanadyl acetylacetonate (VAC) for their potential to stimulate bone formation by mimicking insulin’s anabolic effects on skeletal tissue. In 2012, Paglia et al. [22] studied VAC treatment on angiogenesis and chondrogenesis during early callus formation in diabetic rats. VAC treatment significantly increased cartilage formation, vascular density, and VEGF-C expression within the fracture callus during the first 7–10 days post injury, resulting in enhanced mineralization and mechanical strength by weeks 3–4 [22]. A subsequent study explored calcium sulfate (CaSO_4_) as a delivery scaffold for VAC in the same model. Local release of VAC from the CaSO_4_ carrier further improved biomechanical strength, cartilage formation, and callus organization without inducing ectopic bone formation, supporting CaSO_4_ as an effective vehicle for targeted vanadium delivery and dose reduction [23]. In 2017, another study evaluated the local delivery of VAC in a diabetic rat femoral fracture model, demonstrating significantly improved mechanical strength, enhanced cortical bridging, and increased cartilage and mineralized tissue formation compared to controls [24]. These findings suggest that VAC promotes bone repair by modulating bone activity and accelerating early callus formation [22,23,24,25,26,27,28]. Several studies have also pointed to the influence of vanadium compounds on B cell, T cell, and immune cell signaling, initiating various signaling cascades, GTPases, and transcription factors [29]. Due to this, alterations in carbohydrate and lipid metabolism, gene expression, and cellular mechanisms, such as proliferation and survival, can occur [25,26,29].

Although vanadium compounds have been studied for their insulin-mimetic properties, the mechanisms by which they influence bone cell function remain incompletely understood. During the 20th century, a few papers were published that demonstrated that vanadium derivatives could modulate osteoblast cell proliferation and differentiation. It has been hypothesized that vanadium compounds influence osteoblast function by inhibiting an acid phosphatase-like phosphotyrosyl protein phosphatase activity specific to osteoblasts [21,30,31,32,33]. However, later work focused more closely on the mechanism of insulin mimicry and less on its relationship to bone healing outcomes. For instance, research using Chinese hamster ovary (CHO) cells demonstrated that vanadyl sulfate (VS) exerts insulin-like effects by activating mitogen-activated protein kinase (MAPK) signaling through a phosphatidylinositol 3-kinase (PI3-K)-dependent pathway. Inhibition of PI3-K and farnesyltransferase significantly reduced VS-induced phosphorylation and activation of key signaling proteins, underscoring PI3-K’s central role in mediating these effects [34]. Other researchers showed that organo-vanadium compounds were more effective than inorganic vanadium salts in improving glucose tolerance and insulin sensitivity in diabetic rodent models, primarily through protein kinase B (Akt) activation [26]. Their subsequent work revealed that vanadium compounds stimulate multiple components of the insulin signaling cascade including tyrosine phosphorylation of insulin receptor substrate-1 (IRS-1), activation of extracellular signal-regulated kinases (ERK1/2), PI3-K, and Akt despite acting independently of insulin receptor tyrosine kinase activity. Notably, vanadium’s effects are closely associated with enhanced IRS-1 phosphorylation, suggesting a receptor bypass mechanism of insulin signal mimicry [25,26,35]. Many studies have also highlighted concerns regarding the potential toxicity of conventional vanadium salts at elevated doses, prompting the exploration of alternative delivery strategies.

In response, researchers have developed vanadyl porphyrin-based two-dimensional metal–organic frameworks, offering a promising therapeutic platform with enhanced biocompatibility and controlled release capabilities. These metal–organic frameworks integrate the bioactivity of vanadium ions with the photodynamic and catalytic properties of porphyrin ligands and offer multifunctional capabilities [36,37]. They can be engineered to release vanadyl ions that modulate bone metabolism while simultaneously generating reactive oxygen species under light stimulation to combat infection and inflammation. This dual-action approach positions them as a next-generation solution for bone regeneration, particularly in complex or compromised clinical environments.

In summary, vanadium has improved glucose transport, glycogen and lipid synthesis while inhibiting gluconeogenesis and lipolysis [25,26]. Crossover between vanadium and insulin signaling pathways relies on the activation of mitogen-activated-protein kinases (MAPKs), extracellular signal-regulated kinase 1/2 (ERK1/2) and p38 MAPK, and phosphatidylinositol 3-kinase (PI3-K)/protein kinase B (PKB) [25,26]. Therefore, we hypothesize that at optimal concentrations, VAC and VOSO_4_ will increase osteoblast proliferation and function by activating the AKT signaling pathway like insulin. This data may provide important information to improve clinical healing outcomes.

## 2. Results

### 2.1. Cellular Proliferation Is Enhanced by Treatment with Vanadium (II) Sulfate (VOSO_4_) and Vanadyl Acetylacetonate (VAC)

**Figure 1:**
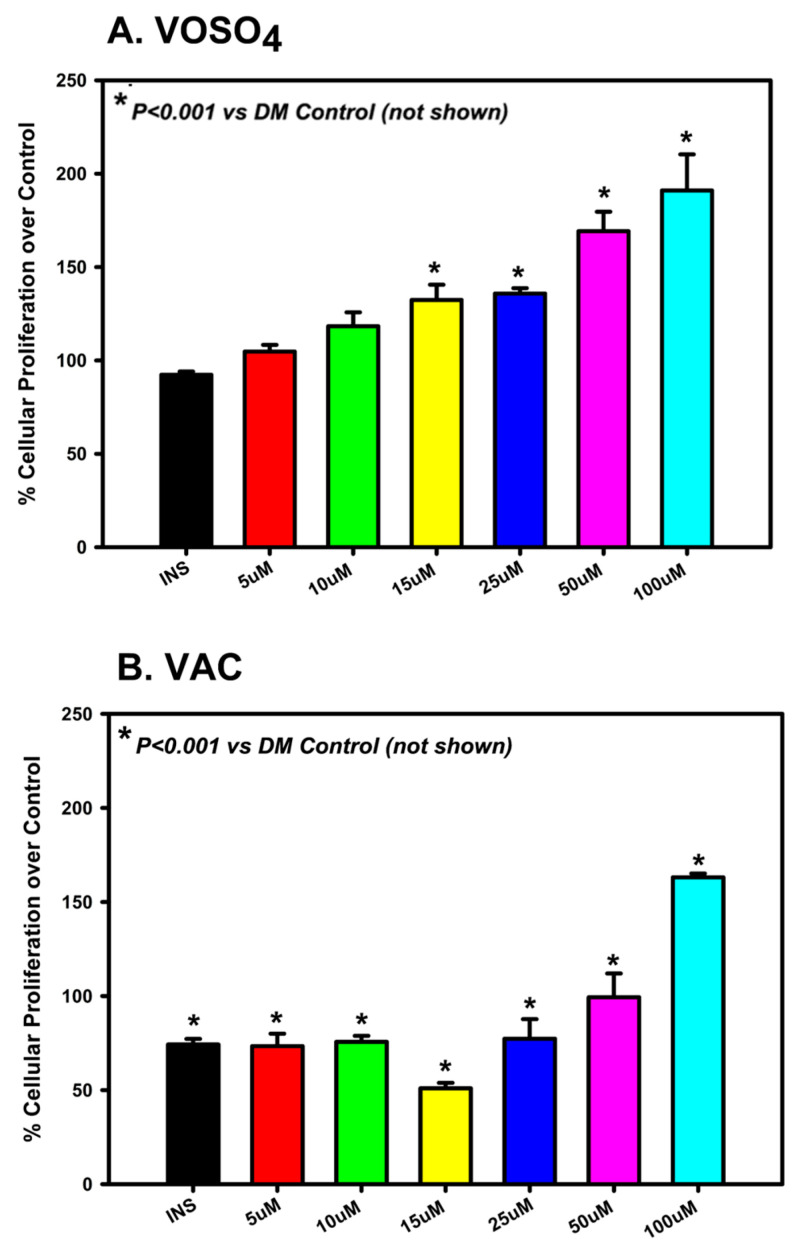
VOSO_4_ (**A**) and VAC (**B**) treatments significantly enhanced proliferation in differentiated MC3T3-E1 cells after 24 h. Experimental groups included cells cultured in differentiation medium (DMC) alone (control), DMC supplemented with 50 µg/mL insulin, or varying concentrations of VOSO_4_ or VAC (n = 3). Data are presented as mean ± SD. Symbols (*) indicate statistically significant differences between treatment groups, with corresponding *p*-values provided.

Osteoblast proliferation in response to VOSO_4_ and VAC treatment was assessed using an MTT assay. Cells were cultured in growth media (DM Control, DMC) and treated for 24 h with either 1% insulin (50 µg/mL) or varying concentrations (0, 5, 10, 15, 25, 50, or 100 µM) of VOSO_4_ or VAC. Treatment with VOSO_4_ at concentrations ranging from 15 µM to 100 µM significantly enhanced osteoblast proliferation compared to the controls (Figure 1A, *p* < 0.001). The most notable rise was observed in the 100 µM VOSO_4_ group, which increased 107% when compared to the DM control. VAC treatment significantly increased cellular proliferation in most groups (*p* < 0.001), except at 15 µM, which showed a significant decrease (*p* < 0.001; Figure 1B). For VOSO_4_-treated groups, the most significant increase was observed at 100 µM VAC, showing a 63% increase compared to the control. In general, increased concentrations of VOSO_4_ and VAC resulted in enhanced cellular proliferation. Rather than assessing proliferation at additional time points, we focused on evaluating the functional effects of these treatments over a 21-day period using Alcian Blue and Alizarin Red staining.

### 2.2. Vanadium (II) Sulfate (VOSO_4_) and Vanadyl Acetylacetonate (VAC) Enhance Extracellular Matrix Mineralization Through Calcium Accumulation

To evaluate the impact of VOSO_4_ and VAC on osteoblast-mediated calcium mineralization, ARS staining was conducted. MC3T3-E1 cells were exposed to increasing concentrations of each compound (0, 25, 50, 100 µM), all supplemented with 1% ascorbic acid and collected for analysis on days 7, 10, 14, 17, and 21 for analysis. These concentrations were categorized as differentiation media control (DMC), low dose (LD), mid dose (MD), and high dose (HD), respectively.

**Figure 2:**
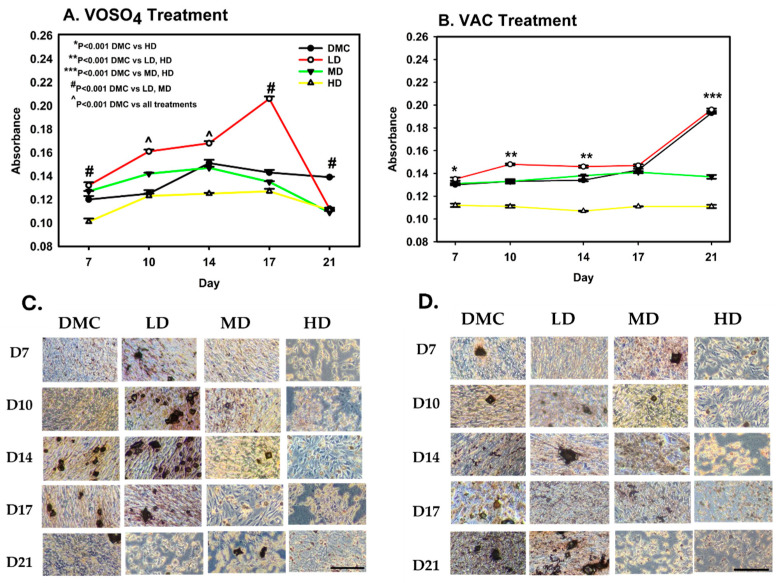
Treatment with VOSO_4_ (**A**,**C**) and VAC (**B**,**D**) enhances calcium mineralization in differentiated MC3T3-E1 cells. Samples were harvested on days 7, 10, 14, 17, and 21 (n = 3). Experimental groups included cells cultured in differentiation medium (DMC) and cells treated with low (LD, 25 µM), medium (MD, 50 µM), or high (HD, 100 µM) doses of VOSO_4_ or VAC, each supplemented with 50 µg/mL AA. Data are presented as mean ± SD. Symbols (*, #, ^) indicate statistically significant differences between groups where applicable; *p*-values are provided. (**C**,**D**) Representative images at 40× magnification. The scale bar in the lower left corresponds to 0.45 mm and applies to all images.

For VOSO_4_, calcium mineralization differed significantly across treatment groups (*p* < 0.001, Figure 2A). By day 7, all treated groups showed statistically significant increases compared to DMC (*p* < 0.05), with the LD and MD groups exhibiting 10% and 16% greater calcium accumulation, respectively (*p* < 0.001). The LD group consistently maintained significantly elevated mineralization from days 10 through 17, peaking on day 17 with a 44% increase relative to DMC.

VAC treatment also resulted in significant differences in calcium deposition among groups (*p* < 0.001, Figure 2B). The HD group showed a marked decrease compared to DMC on day 7 (*p* < 0.001). By day 10, the LD group increased mineralization by 11% when compared to DMC (*p* < 0.001). However, the HD group remained 17% suppressed (*p* < 0.001). Mineralization in the LD group approximated control levels by day 17. However, by day 21, both MD (29% reduction) and HD (42% reduction) groups exhibited substantial reductions in calcium deposition when compared to DMC (*p* < 0.001).

### 2.3. Vanadium (II) Sulfate (VOSO_4_) and Vanadyl Acetylacetonate (VAC) Stimulate Calcium Accumulation in the Extracellular Matrix

**Figure 3:**
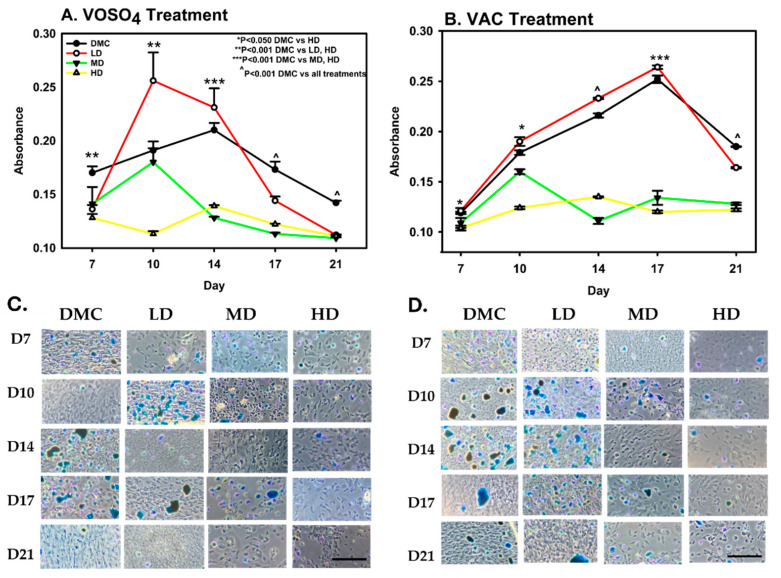
Exposure to VOSO_4_ (**A**,**C**) and VAC (**B**,**D**) enhances proteoglycan accumulation in differentiated MC3T3-E1 cells. Samples were collected on days 7, 10, 14, 17, and 21. Experimental groups included cells cultured in differentiation medium (DMC) or treated with low (LD, 25 µM), medium (MD, 50 µM), or high (HD, 100 µM) concentrations of VOSO_4_ or VAC, each supplemented with 50 µg/mL ascorbic acid. Data are expressed as mean ± SD. Symbols (*, ^) indicate statistically significant differences between groups where applicable; corresponding *p*-values are provided. (**C**,**D**) Representative images at 40× magnification. The scale bar in the lower left corner represents 0.45 mm and applies to all images.

Proteoglycan deposition in MC3T3-E1 cells was measured following treatment conditions identical to those used in the ARS assay. On day 7, VOSO_4_-treated cells exhibited a significant increase in proteoglycan production in both the low-dose (LD) and high-dose (HD) groups compared to the DMC group (*p* < 0.001, Figure 3A). Specifically, the LD VOSO_4_ treatment enhanced proteoglycan synthesis by 1.4-fold relative to controls (*p* = 0.001).

On day 10, low-dose treatment increased proteoglycan deposition, while higher doses resulted in a reduction. By day 14, proteoglycan synthesis in the low-dose group continued to show an upward trend compared to the DMC control. However, the comparison of the DMC group with MD and HD VOSO_4_ groups found significant impairment in proteoglycan deposition levels. (*p* < 0.001). Between days 17 and 21, proteoglycan accumulation in the low-dose treatment group was significantly reduced by 16% compared to the DM control group.

In VAC-treated cells, the high-dose (HD) group exhibited a 13% reduction in proteoglycan deposition compared to the DMC group on day 7. By day 10, the HD group showed a significant 31% decrease relative to DMC (*p* < 0.001), whereas the low-dose (LD) group demonstrated a 6% increase in proteoglycan accumulation (Figure 3B). On day 14, proteoglycan levels in the LD group were 8% higher than the control, while the HD group experienced a 38% decline. By day 21, proteoglycan synthesis decreased in both the DMC and LD groups; however, these groups maintained significantly higher proteoglycan levels compared to the medium-dose (MD) and HD groups (*p* < 0.001).

### 2.4. Impact of Vanadium (II) Sulfate (VOSO_4_) and Vanadyl Acetylacetonate (VAC) on p-Akt Protein Expression

**Figure 4:**
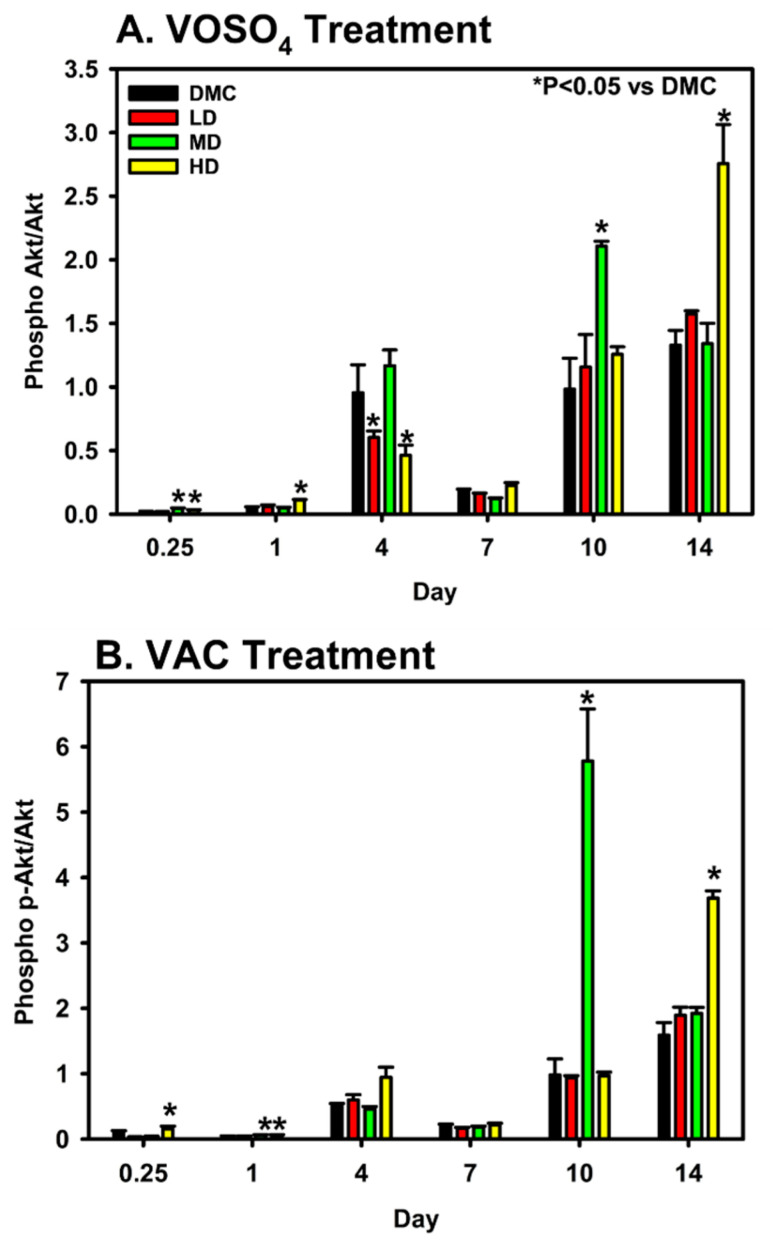
Modulation of p-Akt/Akt expression by VOSO_4_ (**A**) and VAC (**B**) in differentiated MC3T3-E1 cells. Cells were treated with differentiation medium (DMC; growth medium + 50 µg/mL ascorbic acid) or VOSO_4_/VAC at low (LD, 25 µM), medium (MD, 50 µM), and high (HD, 100 µM) doses, all supplemented with 50 µg/mL ascorbic acid. Samples were collected at 0.25, 1, 4, 7, 10, and 14 days post-treatment (n = 3). Data represent mean ± SD. Statistical significance between groups is indicated by * with corresponding *p*-values noted.

Expression levels of total Akt and its phosphorylated, activated isoform, p-Akt, were analyzed in MC3T3-E1 cells following administration of varying doses of VOSO_4_ and VAC. For VOSO_4_-treated cells, the data demonstrates that LD VOSO_4_ results in expression patterns like the control at most timepoints (Figure 4, Days 0.25, 1, 7–14). However, MD VOSO_4_ pAKT/AKT expression is either at a similar level to the DM control (Days 1, 7, 14) or significantly elevated (Days 0.25, 10, *p* < 0.050). HD VOSO_4_ also significantly increased pAKT/AKT at several timepoints (Day 0.25, 1, 14, *p* < 0.05).

For VAC-treated cells, LD VAC-treated cells did not alter pAKT/AKT expression levels. However, both HD and MD-treated cells did show significantly elevated pAKT/AKT expression levels on days 1 and 10, while the HD VAC dose significantly increased on days 0.25, 1, and 14.

### 2.5. Modulation of MC3T3-E1 Cell Gene Expression by Vanadium (II) Sulfate and Vanadyl Acetylacetonate

**Figure 5:**
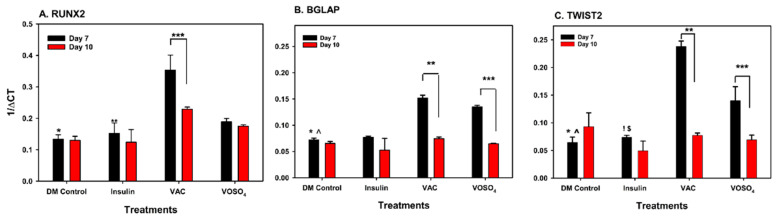
VOSO_4_ and VAC treatments regulate gene expression in differentiated MC3T3-E1 cells. (**A**) * *p* < 0.001 DMC vs. VAC, ** *p* < 0.001 insulin vs. VAC, *** *p* VAC Day 7 vs. Day 10. (**B**) BGLAP, * *p* < 0.001 DMC vs. VAC, ^^^ *p* = 0.003 DMC vs. VOSO_4_, ** *p* < 0.001 VAC day 7 vs. day 10, *** *p* < 0.001 Day 7 vs. Day 10 (**C**) TWIST2, * *p* < 0.001 DMC vs. VAC, ^ *p* < 0.001 DMC vs. VSO_4_, ! *p* < 0.001 Ins vs. VAC, $ *p* = 0.002 vs. VOSO_4_, ** *p* < 0.001 VAC day 7 vs. Day 10, *** *p* < 0.001 VOSO_4_ day 7 vs. day 10. Cells were harvested at days 7, 10, 14, 17, and 21 following treatment. Experimental groups included cells cultured in differentiation medium (DM Control), comprising growth medium supplemented with 50 µg/mL ascorbic acid (AA), or low-dose (LD, 25 µM) VOSO_4_ and VAC treatments, both supplemented with 50 µg/mL AA. Data are presented as mean ± SD. Symbols denote statistically significant differences between groups where applicable.

The effects of vanadium compounds on osteoblast cell line, MC3T3-31, were examined on Days 7 and 10, particularly through the gene expressions of RUNX2, BGLAP, ACP5, AKT and TWIST2. VAC displayed a significant increase in RUNX2 activity on Day 7 relative to control and insulin-treated groups (*p* < 0.001, Figure 5A). A significant decrease in RUNX2 expression between Days 7 and 10 was also noted for the VAC-treated samples (*p* < 0.001). VAC treatment significantly increases (*p* < 0.001) the BGLAP expression on Day 7 when compared to the control (Figure 5B). A significant decline was observed between Day 7 and Day 10 in cells treated with VAC. Similarly, BGALP expression was significantly upregulated by VOSO_4_ relative to control at Day 7. (*p* = 0.003). For ACP5/AKT, vanadium compound treatment demonstrated no significant effect on expression Days 7 and 10. However, ACP was found to have increased expression post VAC or VOSO_4_ treatment when compared to DMC group on day 7. On Day 7, VAC and VOSO_4_ treatment showed a significant increase between control and insulin in TWIST2 expression (Figure 5C) on day 7 (*p* < 0.001) which decreased by day 10.

## 3. Discussion

Recent research has demonstrated that vanadyl acetylacetonate (VAC) promotes bone growth both in vivo and in vitro, indicating its potential as a therapeutic agent for fracture repair [22,23,24]. These studies further demonstrated that intramedullary administration of VAC at fracture sites significantly elevated bone and cartilage formation relative to controls in non-diabetic rodent models [22,23,24]. Nevertheless, the precise cellular and molecular mechanisms by which small-molecule insulin mimetics such as VAC, ZnCl_2_, and VOSO_4_ accelerate bone repair remain inadequately characterized. Emerging data suggest that these compounds promote skeletal restoration by modulating three critical processes: stimulating chondroprogenitor differentiation and osteoblast lineage commitment while concurrently inhibiting osteoclastogenesis [38,39].

Vanadium compounds have been shown to stimulate critical nodes within the insulin signaling pathway including mitogen-activated protein kinases (MAPKs) and ERK1/2 [34], which upregulate vascular endothelial growth factor (VEGF) expression and facilitate neovascularization [23,40]. Additionally, vanadium has been shown to increase tyrosine phosphorylation of insulin receptor substrate-1 (IRS-1) and activate phosphatidylinositol 3-kinase (PI3K), key components of the insulin signaling pathway that contribute to the promotion of bone regeneration [34]. Previous research has demonstrated that VAC activates signaling pathways analogous to those triggered by insulin [41,42,43], suggesting its potential, along with other insulin mimetics, to promote bone repair. However, the precise mechanisms by which VAC facilitates osteogenesis remain incompletely understood, and to date, the impact of VOSO_4_ on bone regeneration has not been thoroughly investigated. In this study, we show that both VAC and VOSO_4_ enhance differentiation of MC3T3-E1 osteoblasts, stimulate calcium and proteoglycan synthesis, and appear to modulate Akt/p-Akt signaling in MC3T3-E1 cells in a biphasic pattern. This pattern begins with initial activation within the first few days, followed by a second phase of induction between days 4 and 14, which mirrors the effects observed with insulin [44].

Our findings indicate enhanced osteoblast proliferation following treatment with insulin-mimetic compounds, aligning with results reported by Ippolito et al., who observed increased bone formation and improved mechanical strength following localized administration at fracture sites [24]. Treatment with VAC and VOSO_4_ significantly promoted extracellular matrix maturation, as evidenced by increased calcium and proteoglycan production. These results are consistent with findings by Sánchez-González et al., where bis(maltolato)oxovanadium(IV) was used to restore impaired calcium mineralization and upregulate osteopontin (OPN) mRNA expression in the femoral tissue of diabetic rat models [45]. Under normal conditions, osteopontin (OPN) binds to osteoclast surfaces, facilitating their adhesion to mineralized bone and contributing to decreased bone density. In diabetic rat models, treatment with bis(maltolato)oxovanadium(IV) has been shown to enhance calcium deposition while downregulating OPN mRNA expression, ultimately restoring bone density to levels comparable to non-diabetic controls [45]. These findings suggest that vanadium-based compounds not only stimulate osteoblast proliferation but also enhance the deposition of calcium and proteoglycans, key components in bone matrix mineralization and hard callus formation. Our data show that calcium and proteoglycan accumulation peaked in MC3T3-E1 cells treated with low concentrations of VAC and VOSO_4_ after 21 days. This trend indicates that although higher concentrations of vanadium compounds may initially promote proliferation, they could compromise long-term cell viability and matrix production.

Based on the literature, VAC and VOSO_4_ should be capable of inducing insulin-induced PI3K signaling [44,46]. Normally, insulin phosphorylates, IRS1 and IRS2, activate the PI3K pathway. Once PI3K is activated, Akt is phosphorylated, forming p-Akt, which activates the transcription factor, FOXO1. Our data indicate that both total Akt and its phosphorylated form (p-Akt) are activated during MC3T3-E1 proliferation in response to VAC and VOSO_4_ treatment in a manner that is both dose- and time-dependent. Runx2 is necessary to promote osteoblast differentiation and TWIST2 often works as a repressor to negatively regulate this process [15]. It is likely that in MC3t3-E1 cells, activation of PI3K/Akt promotes osteogenesis by upregulating and stabilizing Runx2 [47,48]. At the same time, TWIST2 suppression would ultimately relieve the repressive effect on Runx2. This would lead to a dual mechanism that shifts the cells towards osteoblast differentiation and mineralization. Evidence demonstrates that Akt directly modulates Runx2 activity in MC3T3-E1 cells through phosphorylation, thereby enhancing osteogenesis. But direct regulation of TWIST2 by Akt in this cell line is less well-characterized [49], but the available data supports the notion that Akt indirectly contributes to TWIST2 downregulation, further promoting the Runx2-driven osteogenic pathway. Our data on day 7 for both vanadium compounds supports this mechanism demonstrating a significant impact on the signaling molecules, Runx2, BGLAP, and TWIST2 [15,50]. Ultimately, it appears that at lower concentrations, these mimetics modulate signaling pathways and enhance osteoblast functions, including calcium and proteoglycan deposition. Future experiments should investigate whether vanadium induces Akt signaling directly or indirectly regulates TWIST2 expression or activity in osteoblasts, as this potential interaction remains unexplored and could reveal new mechanistic insights into osteogenic differentiation.

These findings are consistent with the following three previous studies [51,52,53]. Gao et al. demonstrated that vanadate (V_2_O_5_) activates hypoxia-inducible factor 1 (HIF-1) signaling by upregulating HIF-1α expression, which subsequently increases VEGF production in DU145 human prostate carcinoma cells [51]. Their study further revealed that vanadate-induced HIF-1α and VEGF expression is dependent on PI3K/Akt pathway activation, with vanadate promoting both PI3K activity and Akt phosphorylation in a dose- and time-dependent manner. In a separate study, Norouzi et al. investigated the insulin-like effects of zinc sulfate (ZnSO_4_), another known insulin mimetic, in mouse (C2C12) and human skeletal muscle cells [53]. ZnSO_4_ independently triggered phosphorylation of key signaling molecules including Akt, tyrosine kinases, GSK-3β, ERK1/2, p38, and SHP-2, mimicking the effects of insulin. Notably, ZnSO_4_-induced Akt phosphorylation occurred at concentrations equivalent to those used for insulin. Finally, Nimmanon et al. focused on the role of zinc transporters, particularly ZIP7 (a member of the SLC39A family), in regulating intracellular zinc levels [52]. ZIP7 facilitates zinc mobilization from intracellular stores such as the endoplasmic reticulum into the cytosol. In MCF-7 human breast cancer cells, zinc treatment of ZIP7-overexpressing cells significantly enhanced ZIP7-mediated zinc release, leading to the activation of downstream signaling pathways including PI3K/Akt, MAPK, and mTOR. These results suggest that ZIP7 plays a central role in zinc-induced activation of proliferative signaling cascades. Together with our findings, these studies support the conclusion that vanadium-based compounds and other insulin mimetics can stimulate cellular proliferation through activation of the PI3K/Akt pathway, closely paralleling insulin’s mechanism of action [54]. Future studies should investigate the comparative and combinatorial effects of insulin mimetics such as vanadate and zinc sulfate on PI3K/Akt-mediated signaling cascades across various cell types, including osteoblasts and other insulin-responsive tissues. In particular, elucidating the role of zinc transporters like ZIP7 in mediating intracellular zinc dynamics and subsequent activation of PI3K/Akt and mTOR pathways could uncover novel mechanisms linking metal ion homeostasis to cellular proliferation, differentiation, and metabolism. Additionally, dissecting how these mimetics influence HIF-1α stabilization and VEGF expression in non-cancerous systems may reveal broader physiological roles for these compounds in angiogenesis and tissue repair.

In summary, our results demonstrate that exposure of MC3T3-E1 pre-osteoblasts to vanadyl acetylacetonate and vanadium (II) sulfate promotes both cellular proliferation and osteogenic function over time. Elevated levels of phosphorylated Akt (p-Akt) relative to total Akt in both treatment groups indicate robust Akt activation via phosphorylation. The sustained expression of p-Akt supports the involvement of the PI3K/Akt signaling cascade in mediating the pro-osteogenic effects of these insulin-mimetic agents, mirroring the mechanism of insulin action. Future studies may explore additional downstream targets of the PI3K pathway such as forkhead box O1 (FOXO1) and the mechanistic target of rapamycin (mTOR) to further delineate the molecular basis through which vanadium compounds facilitate skeletal regeneration and matrix mineralization.

## 4. Materials and Methods

### 4.1. Cell Culture

MC3T3-E1 pre-osteoblast cells were generously provided by the O’Connor Laboratory (Rutgers New Jersey Medical School, Newark, NJ, USA). Cells were cultured in growth medium composed of Minimum Essential Medium (MEM; Corning, Corning, NY, USA) supplemented with 5% fetal bovine serum (FBS; Atlanta Biologicals, Miami, FL, USA), 1% penicillin–streptomycin (Corning, Corning, NY, USA), 1% L-glutamine (Corning, Corning, NY, USA), and 1% sodium pyruvate (Corning, Corning, NY, USA). Experimental treatments included insulin (Invitrogen, Carlsbad, CA, USA), vanadium (II) sulfate (VOSO_4_; Sigma-Aldrich, St. Louis, MO, USA), and vanadyl acetylacetonate (VAC; Sigma-Aldrich, St. Louis, MO, USA). Cultures were maintained at 37 °C in a humidified incubator with 5% CO_2_ and subcultured upon reaching 75–80% confluency. Cells were plated at densities appropriate to each experimental condition. Treatment groups included vehicle controls (1% ascorbic acid at 50 µg/mL and 1% insulin at 10 µg/mL) as well as VAC and VOSO_4_ at concentrations of 0 µM, 5 µM, 10 µM, 15 µM, 25 µM, 50 µM, and 100 µM. Both insulin and ascorbic acid served as experimental controls for osteogenic differentiation and proliferative activity [19].

### 4.2. Cell Proliferation Assessment

Cell proliferation was quantified using an MTT assay kit (Abcam, Cambridge, UK). MC3T3-E1 cells were seeded at a density of 8000 cells per well and treated for 24 h with 1% insulin (control), or varying concentrations (0 µM, 5 µM, 10 µM, 15 µM, 25 µM, 50 µM, and 100 µM) of vanadyl acetylacetonate (VAC) or vanadium (II) sulfate (VOSO_4_). Following treatment, the MTT reagent was applied according to the manufacturer’s protocol. Formazan product formation was measured by recording absorbance at 590 nm using a 96-well plate reader (SpectraMAX M5, Molecular Devices, San Jose, CA, USA).

### 4.3. Calcium Deposition Assay

Calcium deposition in treated cell cultures was evaluated using the anthraquinone dye Alizarin Red S (ARS). Post treatment, cells were fixed with 4% paraformaldehyde for 15 min at ambient temperature. Following fixation, cells were rinsed twice with deionized water (diH_2_O) before incubation with 40 mM ARS solution for 20 min at room temperature with gentle agitation. Excess dye was removed by washing the wells four times with diH_2_O, and stained cultures were imaged using phase-contrast microscopy. For quantification of calcium deposits, 10% acetic acid was added to each well and incubated at room temperature with gentle shaking for 30 min to solubilize the dye-bound calcium. The resulting acetic acid–dye solution was collected into microcentrifuge tubes, vortexed for 30 s, heated at 85 °C for 10 min, then rapidly cooled on ice for 5 min. Samples were centrifuged at 13,500× *g* for 20 min to pellet debris. The clarified supernatant was neutralized with 10% ammonium hydroxide, transferred to a 96-well plate, and absorbance was measured at 405 nm using a SpectraMAX M5 microplate reader (Molecular Devices, San Jose, CA, USA).

### 4.4. Proteoglycan Deposition Assay

Proteoglycan deposition was assessed using Alcian blue staining. Cells were fixed with ice-cold 100% methanol for 5 min, followed by overnight incubation at 4 °C in 0.1% Alcian blue solution prepared in 0.1 M HCl. After staining, cells were rinsed three times with 1X Hank’s Balanced Salt Solution (HBSS), and images were captured using phase-contrast microscopy. For quantification, proteoglycans were extracted by incubating the stained cells with 6 M guanidine-HCl at room temperature, followed by overnight storage at 4 °C. The resulting extracts were transferred to a 96-well microplate, and absorbance was measured at 595 nm using a SpectraMAX M5 microplate reader (Molecular Devices, San Jose, CA, USA).

### 4.5. Immunoblotting

Protein expression levels of Akt and phosphorylated Akt (p-Akt) were evaluated by immunoblotting in triplicate. After treatment, cells were rinsed with Hank’s Balanced Salt Solution (HBSS) and lysed using Mammalian Protein Extraction Reagent (M-PER; GE Healthcare, Chicago, IL, USA), followed by gentle rocking at room temperature for 5 min. Lysates were then centrifuged at 13,500× *g* for 20 min, and the clarified supernatant was collected for analysis. Protein samples were prepared by mixing equal volumes of lysate and 2X SDS-PAGE sample buffer containing 10% β-mercaptoethanol (GE Healthcare, Chicago, IL, USA). Samples were heated to 70 °C and resolved on 4–12% gradient NuPAGE Bis-Tris gels (Thermo Fisher Scientific, Waltham, MA, USA) according to the manufacturer’s instructions. Separated proteins were transferred onto polyvinylidene fluoride (PVDF) membranes, which were blocked overnight at 4 °C with SuperBlock blocking buffer (Thermo Fisher Scientific, Waltham, MA, USA) to reduce nonspecific binding. Primary immunodetection was performed using mouse- and rabbit-derived antibodies against GAPDH, total Akt, and p-Akt (all from Cell Signaling Technology, Danvers, MA, USA). Secondary detection employed horseradish peroxidase (HRP)-conjugated anti-mouse and anti-rabbit antibodies (R&D Systems, Minneapolis, MN, USA) corresponding to the primary antibodies. Protein bands were visualized via enhanced chemiluminescence (ECL) following the manufacturer’s protocol. Chemiluminescent signals were captured and quantified using a ProteinSimple FluorChem E system (ProteinSimple, San Jose, CA, USA) and analyzed with AlphaView software (Cell Biosciences Inc., Santa Clara, CA, USA Version 3.3.1.0).

### 4.6. Quantitative PCR

RNA samples were collected from cultured MC3t3 cells treated with LD VAC and VOSO_4_. For each time point and treatment group, three independent samples were collected. Prior to RNA isolation, culture media were aspirated and cells washed three times with 1X HBSS. Total RNA was extracted immediately from MC3T3-E1 cells using the RNeasy Mini Kit (Qiagen, Gaithersburg, MD, USA) following the manufacturer’s instructions, and RNA was eluted in 20 µL of RNase-free water. RNA concentration was measured spectrophotometrically using a Biodrop (Biodrop), and quality was assessed by A260/A280 ratios (acceptable range: 1.8–2.0) and integrity verified via agarose gel electrophoresis. Only samples meeting these criteria were used for cDNA synthesis. RNA samples were stored at −80 °C until use.

For cDNA synthesis, 1 µg of total RNA was reverse transcribed in a 20 µL reaction containing 1X RT buffer, 0.5 mM dNTPs, 1 µL RNase inhibitor, and 1 µL M-MLV reverse transcriptase (all from New England BioLabs, Ipswich, MA, USA). Reactions were incubated at 42 °C for 1 h, followed by enzyme inactivation at 95 °C for 10 min. Resulting cDNA was stored at −20 °C.

Quantitative PCR (qPCR) was performed in triplicate using SYBR^®^ Green Master Mix (Thermo Scientific, Waltham, MA, USA) in 96-well plates. Primer sequences (Table 1) were designed to amplify 100–130 bp products. Each 25 µL reaction contained 500 nM ROX dye, 3 mM MgCl2, 70 nM forward and reverse primers, and 250 ng template cDNA. qPCR was run on an ABI StepOnePlus System (Applied Biosystems, Waltham, MA, USA) with the following cycling conditions: initial denaturation at 95 °C for 15 min, 40 cycles of 95 °C for 15 s, 58–60 °C for 30 s, and 72 °C for 60 s, followed by a melt curve analysis. Data were analyzed using Applied Biosystems Sequence Detection Software v2.3 employing the comparative Cq method. Negative controls (no template control, NTC) and positive controls were included on each plate to ensure assay integrity and detect inter-plate variation. Gene expression was considered valid for Cq values between 15 and 35; values ≥ 40 were excluded. Triplicate Cq values were normalized to the reference gene GAPDH, verified to be stable under chondrogenic conditions. Relative expression levels were calculated using the 2^ (-ΔCq) method.

## Figures and Tables

**Table 1:** Real-Time QPCR Experiment Primer Sequences.

Oligo Name	Sequence
B-Actin_R	GACAGGATGCAGAAGGAGATTACTG
B-Actin_F	CCACCGATCCACACAGAGTACTT
ACP5_F	TGAGGACGTATTCTCTGACCG
ACP5_R	CACATTGGTCTGTGGGATCTTG
BGLAP_F	GGCGCTACCTGTATCAATGG
BGLAP_R	GTGGTCAGCCAACTCGTCA
TWIST2_F	CGCTACAGCAAGAAATCGAGC
TWIST2_R	GCTGAGCTTGTCAGAGGGG
RUNX2_F	GACGAGGCAAGAGTTTCACC
RUNX2_R	GGACCGTCCACTGTCACTTT

## Data Availability

The dataset is available on request from the authors.

## Abbreviations

The following abbreviations are used in this manuscript:

AA	Ascorbic Acid
ACP5	Acid Phosphatase 5, Tartrate Resistant
Akt	Protein Kinase B
ARS	Alizarin Red Staining
BGLAP	Bone Gamma-Carboxyglutamate Protein (Osteocalcin)
cDNA	Complementary DNA
Cq	Quantification Cycle (Threshold Cycle in qPCR)
DMC	Differentiation Medium Control
DMEM	Dulbecco’s Modified Eagle Medium (or MEM as used)
ER	Estrogen Receptor
ERK1/2	Extracellular Signal-Regulated Kinase 1/2
FBS	Fetal Bovine Serum
FOXO1	Forkhead Box Protein O1
GAPDH	Glyceraldehyde 3-phosphate Dehydrogenase
HBSS	Hanks’ Balanced Salt Solution
HD	High Dose
HRP	Horseradish Peroxidase
IL-1	Interleukin-1
IRS	Insulin Receptor Substrate
LD	Low Dose
MAPK	Mitogen-Activated Protein Kinase
MC3T3-E1	Mouse Calvaria Pre-Osteoblast Cell Line
MD	Medium Dose
MEM	Minimum Essential Medium
MMP	Matrix Metalloproteinase
MPER	Mammalian Protein Extraction Reagent
MTT	Methylthiazolyldiphenyl-tetrazolium Bromide
NTC	No Template Control
OPN	Osteopontin
p-Akt	Phosphorylated Akt
PCR	Polymerase Chain Reaction
PI3K	Phosphatidylinositol 3-Kinase
qPCR	Quantitative PCR
RNA	Ribonucleic Acid
RT	Reverse Transcription
RUNX2	Runt-related Transcription Factor 2
SDS-PAGE	Sodium Dodecyl Sulfate Polyacrylamide Gel Electrophoresis
TGF-β	Transforming Growth Factor Beta
TNF-α	Tumor Necrosis Factor Alpha
TWIST2	Twist-Related Protein 2
VAC	Vanadyl Acetylacetonate
VEGF	Vascular Endothelial Growth Factor
VOSO_4_	Vanadium (IV) Oxide Sulfate
